# What's in a Name? Etymology and History of Antibacterials and Antifungals

**DOI:** 10.1093/ofid/ofag440

**Published:** 2026-08-13

**Authors:** Chad Beisel, Monica Donnelley, Pegah Capaul, Tanya Savenka, Ashly Nham, Matthew Gosen, James Lewis, Michael Barbachyn, Angel N Desai, George R Thompson

**Affiliations:** Department of Pharmacy, University of California–Davis Medical Center, Sacramento, California, USA; Department of Pharmacy, University of California–Davis Medical Center, Sacramento, California, USA; Department of Pharmacy, University of California–Davis Medical Center, Sacramento, California, USA; Division of Infectious Diseases, Department of Internal Medicine, University of California–Davis Medical Center, Sacramento, California, USA; Department of Pharmacy, University of California–Davis Medical Center, Sacramento, California, USA; Division of Infectious Diseases, Department of Internal Medicine, University of California–Davis Medical Center, Sacramento, California, USA; Department of Pharmacy, University of Oregon Health Sciences University, Portland, Oregon, USA; Barbachyn Consulting, LLC, Kalamazoo, Michigan, USA; Division of Infectious Diseases, Department of Internal Medicine, University of California–Davis Medical Center, Sacramento, California, USA; Division of Infectious Diseases, Department of Internal Medicine, University of California–Davis Medical Center, Sacramento, California, USA; Department of Medical Microbiology and Immunology, University of California–Davis, Davis, California, USA

**Keywords:** antibacterials, antifungals, antimicrobials, etymology, history

## Abstract

The development of antimicrobials represents some of the most storied and transformative scientific milestones of the modern era. This review provides an overview of their historical development and etymologic roots, highlighting their enduring contributions to contemporary medicine and public health.

Antimicrobials represent one of the most significant scientific breakthroughs in modern medicine, dramatically reducing mortality from infectious diseases [1]. The use of antimicrobial compounds dates to antiquity; natural remedies, such as honey and moldy bread applied to wounds, represent early attempts at biocidal treatment [2]. Honey was valued for its healing properties by ancient civilizations among the Sumerians, Egyptians, and Greeks [3]. Its antimicrobial effects—due to acidity, low moisture, and constituents including hydrogen peroxide—have since been confirmed [4]. Ancient manuscripts also describe placing moldy bread on wounds in Egypt, foreshadowing the later discovery of penicillins and other antimicrobials obtained from fungi [3].

The scientific foundation for antimicrobial therapy was presented in the early 20th century with Dr Paul Ehrlich's “magic bullet” concept, where a therapeutic agent (the “bullet”) would attack and/or kill only specific pathogens or cell types. His work culminated in the development of the first antimicrobial agent, arsphenamine (Salvarsan), which was used for the treatment of syphilis [5]. The discovery of sulfonamides in the 1930s and penicillin in the 1940s heralded the antibiotic era, enabling effective treatment of once-deadly infections and facilitating advances in surgical care, immunosuppressive therapies, and intensive care medicine. The term “antibiotic” was introduced by Selman Waksman in 1941 to describe naturally produced microbial substances that inhibit the growth and/or kill microorganisms [6]. Although originally limited to naturally derived compounds, the term now encompasses natural and synthetic agents with antimicrobial activity and ushered in the “arms race” in clinical medicine between antimicrobials and resistance that continues today.

Understanding the development and mechanisms of modern antibacterials and antifungals is essential for confronting current challenges in infectious disease treatment. This review examines the historical aspects and naming of currently available agents. Antivirals and antimycobacterial agents are not reviewed here.

Naming of agents was traditionally related to aspects of their discovery or molecular structure, while more recent naming conventions continue the same suffixes for new agents in the same class, with the prefix determined by processes developed by the United States Adopted Names Council (USAN Council) [7] in coordination with the World Health Organization's International Nonproprietary Names Programme [8] to ensure global consistency. Prefixes can be proposed by drug development companies, but the name must distinguish the new drug from other currently available agents to avoid confusion; these generally contain 2 syllables to aid in distinguishing them from other agents, do not promise a particular outcome (eg, “Cureall”), and generally avoid medical terminology or the letters H, K, J, W, and Y as they are not used by some Roman-based languages in their alphabets. Considerations for naming also include their potential interpretation when viewed in other languages (eg, the prefix “nova-” is “no go” in Spanish), and ease of pronounceability [9]. By tracing the origins of these agents and exploring how they have shaped clinical practice, we gain critical insights into the triumphs and vulnerabilities of antimicrobial discovery and use.

## PENICILLINS

The name “penicillin” originates from the fungus *Penicillium notatum* (later identified as *P rubens* by whole genome sequencing [10]), discovered by Alexander Fleming in 1928 when he noted the antibacterial properties of the fungus with inhibition of staphylococci surrounding mold growth on culture media [11]. “Penicillin” initially referred to the specific compound isolated from this mold; however, subsequent investigation revealed several penicillins that differed in chemical composition. They were found to contain a common nucleus and differ in their side chains (-R), and the term “penicillin” was used to thereafter denote an entire class of antibiotics sharing a core β-lactam ring structure [5]. Originally identified using Roman numerals in England, they were designated sequentially by letters within the United States until ultimately named by prefixes defining the chemical nature of the side chain “-R.” Penicillin G retained the sequential letter designation (also called benzylpenicillin or penicillin II in the United Kingdom numbering system) and was named in this fashion, but penicillin V did not receive its naming by this convention [12]. Penicillin V was named after *Vertraulich*, the German word for confidential or secret, while the K in “PenVK” refers to its potassium salt form [13]. Penicillins advancing to clinical trials were chosen primarily by their efficacy against Gram-positive pathogens and syphilis, both major areas of unmet need.

Over time, penicillins have been chemically modified to overcome resistance and broaden their spectrum of activity, leading to the development of second-generation penicillins (penicillinase-resistant penicillins such as methicillin), third generation (aminopenicillins such as amoxicillin), and fourth generation (such as carboxypenicillins and ureidopenicillins) (Table 1) [14]. These structural changes (amino side chain in amoxicillin or ureido side chain in piperacillin) have enhanced their activity against Gram-positive and Gram-negative bacteria, while maintaining their mechanism of bacterial cell wall synthesis inhibition via penicillin-binding proteins. Fleming's 1928 discovery of penicillin marked a turning point in medical history, but it was not until more than a decade later that additional investigation was undertaken by Howard Florey and colleagues that led to widescale penicillin manufacture and clinical availability.

**Table 1.** Penicillins

Penicillin	Penicillin was named after the *Penicillium* mold from which it was initially isolated. The name is derived from the Latin word *penicillus*, which means “paintbrush,” according to the shape of the mold’s conidiophores. In chemistry, the suffix “-in” denotes a material, specifically a chemical compound or active ingredient.	[15]
Dicloxacillin	Dicloxacillin is a semisynthetic penicillin whose name reflects its structure: the prefix “di-” indicates the presence of 2 chlorine atoms, while “cloxacillin” refers to its derivation from oxacillin, sharing the isoxazolyl side chain that provides resistance to penicillinase.	[16, 17]
Nafcillin	“Nafcillin” comes from its chemical structure and development procedure. The “naf-” prefix most likely alludes to the compound's naphthalene-derived structure, as it is a naphthalene-based penicillin analogue. The suffix “-cillin” is a common identification for penicillin-related antibiotics.	[18]
Oxacillin	The name “oxacillin” originates from its chemical structure and relationship to other penicillin antibiotics. The “oxa” prefix refers to the oxygen atom in the β-lactam ring of the chemical.	[19]
Flucloxacillin	Another semisynthetic penicillin whose name reflects its structure: a fluorinated and chlorinated derivative of oxacillin.	[20]
Amoxicillin	Amoxicillin's name is derived from a mixture of its chemical components. The “amox-” component of the name refers to the hydroxy-amino-benzyl group added to the penicillin structure, which distinguishes it from previous penicillins.	[21]
Ampicillin	The term “ampicillin” comes from a combination of “am-” for amino (identifying the amino group in its chemical structure) and “-p-,” a bridge representing the phenyl- or benzyl- group (from alpha-amino benzyl), and “-icillin” (marking its origin as a member of the penicillin family of antibiotics).	[22]
Ticarcillin	Ticarcillin was named after the thiophene, a sulfur-containing ring (tic-), with “-ar-,” a linking element with no stand-alone meaning, and “-cillin” after the penicillin class.	[23]
Piperacillin	The name “piperacillin” comes from the chemical components of the medicine. The “piper-” prefix alludes to the presence of a piperazine ring, which contributes to the compound's increased effectiveness against a range of bacteria. The “-cillin” suffix is unique to the penicillin class.	[24]

Improvements in the production of penicillin occurred during World War II through a joint effort between the United States and Britain, which focused on improving production methods. A key breakthrough came with the identification of a different species producing higher yields (*Penicillium chrysogenum*) and the use of corn steep liquor, a nutrient-rich by-product of corn processing, as an effective growth medium [25]. The beer industry played a pivotal role by applying its fermentation expertise to develop deep tank fermentation, enabling penicillin to be produced in quantities sufficient for military use by 1943. Although Fleming is often solely credited, the essential contributions of Florey and Ernst Chain were recognized with a shared Nobel Prize in 1945. Importantly, associated industrial scientists were crucial in large-scale production bringing penicillin to clinical use [26]. By 1943, penicillin was available in sufficient quantities for Allied forces. Secrecy surrounding penicillin's production was crucial during World War II, and German officials requested Fleming's strain from the Institut Pasteur in France but were instead given a strain that did not produce penicillin. However, information leaks continued, and Fleming himself had sent a culture of *Penicillium* strains to Dr H. Schmidt in Germany in the 1930s. X-ray crystallography elucidated the structure of penicillin in 1945, enabling chemical modifications and assessment of derivatives of this agent and widescale use [27].

## β-LACTAMASE INHIBITORS

The first β-lactamase enzyme was identified prior to the clinical use of penicillin in *Bacillus* (later named *Escherichia coli*) and coined “penicillinase” by Abraham and Chain (Table 2) [28, 29]. The clinical relevance of this was not immediately recognized given the focus of penicillin as an anti-*Staphylococcus* and anti-*Streptococcus* medication. However, penicillinases were soon thereafter discovered in other bacteria, prompting the search for agents to inactivate this enzyme [29]. Clavulanic acid was isolated from *Streptomyces clavuligerus* in the 1970s [30, 36]. The structural similarity between the penicillin and clavulanic acid nuclei likely explains the affinity of this compound for some β-lactamases. Subsequent work focused on the development of derivatives of penicillanic acid with sulbactam and tazobactam examples of rational drug design in this area. Combining β-lactamase inhibitors with β-lactam agents improved their reliability in the treatment of select Gram-positive and Gram-negative pathogens.

**Table 2.** β-Lactamase Inhibitors

Clavulanic acid	The first β-lactamase inhibitor was isolated from *Streptomyces clavuligerus* during the investigation of metabolites in culture filtrate. Clavulanate (the salt form of the acid in solution) has little antimicrobial activity on its own but, when combined with other agents, results in significant reductions in minimum inhibitory concentration values against some pathogens.	[29, 30]
Sulbactam	Sulbactam is a halogenated derivative of penicillanic acid (penicillanic acid sulfone). “Sulb-” comes from sulfone, while “-bactam” is a contraction used for β-lactamase inhibitor.	[22]
Tazobactam	An analogue of sulbactam, the “tazo-” prefix is derived from the triazolylmethyl group.	[31]
Vaborbactam	Boronic acid–containing compounds were developed for their selectivity toward β-lactamases over mammalian serine proteases. The “Va-” prefix was added from the medication’s brand name and chosen for pronouncability similar to other more recent naming conventions.	[32]
Relebactam	The etymology of this agent is not publicly documented, although some sources have proposed it as “reliever” creating the “rel-” prefix.	[33]
Avibactam	The original developer named the compound AVE1330A during preclinical development. “Avi-” likely derives from this early name.	[34]
Durlobactam	The etymology of this agent is not publicly documented and likely reflects more recent naming conventions favoring pronounceability.	[35]

## CEPHALOSPORINS

Cephalosporins are broad-spectrum β-lactam antibiotics and are relatively resistant to penicillinases. They were originally derived from the filamentous fungus *Acremonium chrysogenum*—previously known as *Cephalosporium acremonium* where they received their name. The first cephalosporin compound, cephalosporin C (the C denoting the order of discovery), was isolated in 1948 by Italian scientist Giuseppe Brotzu from sewage in Sardinia after noting that it prevented typhoid fever in swimmers [37, 38]. Cephalothin is a semisynthetic derivative of cephalosporin C and acquired its name by the thiophene (“-th”) group added to the cephalosporin compound. Over time, cephalosporins have been additionally modified and advanced through several generations, each changing its effectiveness against a range of bacteria and its susceptibility profile to clinically relevant β-lactamases (Table 3). The initial agents were primarily active against Gram-positive bacteria, while later generations were developed to tackle growing resistance rates in Gram-negative bacteria and other problematic pathogenic bacteria.

**Table 3.** Cephalosporins

Cefazolin	Cefazolin is a first-generation cephalosporin produced from cephalosporin C, a natural antibiotic discovered in 1945 from a mold in Sardinia, Italy. Over time, cephalosporin C underwent several chemical alterations, eventually resulting in the discovery of 7-aminocephalosporanic acid in 1962, which served as the fundamental structure for several later-generation cephalosporins. “Cef-“ refers to the cephalosporin class, while “-azol-” refers to the thiadiazole ring and “-in” a common addition for antimicrobial naming.	[39]
Cephalexin	Cephalexin, another first-generation cephalosporin antibiotic, was licensed by the US Food and Drug Administration in 1970. “Ceph-” refers to the cephalosporin class, while “-alexin” is less clear with no sources definitely describing the naming. However, “alexin” is Greek for “ward off” and an antiquated term for “complement,” and it is likely that this was chosen as a cephalosporin to “ward off” infections.	[40]
Cefadroxil	Cefadroxil is a semisynthetic cephalosporin antibiotic that was created as an oral alternative to previous cephalosporins. The “-adroxil” suffix refers to a hydroxylated side chain.	[41]
Cefuroxime	The name “cefuroxime” combines its cephalosporin origin and unique chemical structure. The “cef-” prefix links it to the cephalosporin class, derived from the mold *Acremonium*. The “-uroxime” part refers to its chemical features: “oxy” represents the oxygen atom in the β-lactam ring, and “-ime” indicates the presence of an oxime group.	[42]
Cefotetan	The name “cefotetan” derives from its classification as a cephamycin, a subset of cephalosporins, and its chemical structure, which includes a 7-methyl-tetrazol-5-yl side chain.	[43]
Cefaclor	Named after the cephalosporin class and chlorine at the 3 position of the cephem nucleus.	[44]
Cefoxitin	Derived from cephamycin C and produced by *Streptomyces lactamdurans*: the suffix “-ox-” signifies the 7-α-methoxyl group, with “-tin” an abbreviation for thiophene.	[45]
Cefprozil	Cefprozil’s suffix is a compound abbreviation of its chemical name with “-pro-” referring to the propenyl group and “-zil” from the benzyl portion of the drug structure.	[46]
Ceftriaxone	Ceftriaxone was developed as a semisynthetic derivative of cephalosporin C, with modifications to enhance its spectrum of activity and pharmacokinetic properties. The suffix is an altered form of the triazine-3-thiol substituent.	[47]
Cefdinir	Cefdinir, like other cephalosporins, is a semisynthetic antibiotic derived from the cephalosporin class. The “-dinir” suffix signifies its particular chemical structure and properties: di-vinyl and n-itroso/i-mine chemical groups present in its structure.	[48]
Cefotaxime	The name “cefotaxime” is derived from its classification and chemical structure. The “-taxime” suffix is an abbreviation of the -thiazolyl and methyloxime-containing side chains.	[49]
Cefixime	The suffix “-ixime” likely refers to a chemical modification made to enhance its stability and oral bioavailability vs earlier cephalosporins. This modification involves the oxime/vinyl group at the 3 position of the cephem nucleus, which facilitates intestinal absorption.	[50]
Ceftazidime	The name “ceftazidime” derives from its chemical structure with “-taz-” referencing the aminothiazolyl side chain and “-idime” the pyridinium structure.	[51]
Cefpodoxime	The “-podoxime” suffix refers to the methoxyimino group. No other information is available regarding naming of this antibacterial.	[52]
Cefepime	The suffix “-epime” references the (methoxyimino) or oxime moiety in its side chain at the C-7 position, which enhances β-lactamase stability.	[53]
Ceftaroline	Derived from chemical modification of cefozopran, this agent was named in similar fashion to other recently developed antimicrobials with the suffix and name selected for pronounceability to denote a different generation of agent from its predecessors.	[54]
Ceftobiprole	Similar to other recently approved medications, the name was chosen to indicate a unique class (eg, different suffix) and for pronounceability.	[55]
Ceftolozane	The suffix is proposed to be a hybridized derivative of its specific chemical structure, which includes a novel oxyimino-aminothiazolyl.	…
Cefiderocol	Similar to other recently approved medications, the name was chosen to indicate a unique class (eg, different suffix) and for pronounceability.	[56]
Ceftibuten	The suffix is derived from the presence of a butanoic acid component in the drug structure.	[57]
Cefditoren	Similar to other approved medications, the name was chosen to indicate a unique class (eg, different suffix) and for pronounceability.	[58]

## CARBAPENEMS

The carbapenems were developed in response to extended-spectrum β-lactamases and have been an essential part of therapy in the treatment of resistant Gram-negative bacteria. Carbapenems were initially developed amid the search for β-lactamase inhibitors in response to the observation of penicillin-resistant organisms in the 1960s. The first carbapenem, thienamycin, was isolated from *Streptomyces cattleya* in the 1970s (“thiena-” refers to the sulfur [thio- or thien-]–containing side chain) [59, 60]. However, thienamycin demonstrated chemical instability that halted its development as a therapeutic option. Despite this, the development of thienamycin prompted the discovery of stable derivatives that retained similar minimum inhibitory concentrations against extended-spectrum β-lactamase–producing pathogens. The carbapenem class was named after the 4:5 fused ring that is shared with penicillins (“penems”), with 2 distinct differences: a substitution of the sulfur atom present in the 1 position of penems with a carbon atom (“carba”) and a double bond between C2 and C3 [61]. The carbapenems also incorporate a unique (1*R*)-1-hydroxyethyl side chain at the C6 position. Carbapenems are named with a prefix based on their unique carbon atom ring substitution and the suffix “-penem” for their unsaturated penem 5-membered ring system (Table 4).

**Table 4.** Carbapenems and Monobactams

Imipenem	The prefix “imi-” may refer to the name *N*-formimidoyl side chain, which contains an imino-like functional group.	[59, 62]
Meropenem	The prefix “mero-” means partial or part. This may refer to the modified structure of the carbapenem ring to include a methyl group at carbon 1 to stabilize the molecule against DHP-1, although this is speculative and references cannot be found.	[61]
Ertapenem	A synthetic carbapenem, there is no information in the public domain regarding the prefix “erta-.” It was likely chosen to reflect the drug class and for pronounceability, as is the convention with more recent drug names.	[63]
Sulopenem	The prefix “sul-” refers to the sulfur atom present in the molecule. Sulopenem is specifically classified as a thiopenem	[64]
Tebipenem	The prefix was likely selected for pronounceability similar to other recently approved antimicrobials.	…
Aztreonam	The “az-” prefixes is derived from the 2-azetidinone ring moiety.	[65]

The first carbapenem that became available for therapeutic use in 1985 was the *N*-formimidoyl amidine derivative of thienamycin, named imipenem, administered as a combination with cilastatin to slow its degradation by renal dehydropeptidase I [62]. Meropenem was developed in the next decade to resolve imipenem's susceptibility to renal metabolism and dependency on the dehydropeptidase I inhibitor cilastatin by appending a sterically bulky methyl group at C1 [61, 66]. The first long-acting carbapenem, ertapenem, was later developed in 2001 with a unique benzoic and carboxylic acid moiety that resulted in increased lipophilicity and net negative charge. The structural changes caused a sizable increase in protein binding to serum albumin and consequential prolongation of the half-life that permitted daily dosing [67].

## MONOBACTAMS

Monobactam refers to a group of compounds that are monocyclic β-lactams and were originally referred to as such by Sykes et al in 1981 [68]. Monobactams do not contain the fused bicyclic thiazolidine and dihydrothiazine ring structures characteristic of penicillins and cephalosporins, respectively. Two novel monobactams, sulfazecin and isosulfazecin, were initially isolated as natural products from 2 species of the genus *Pseudomonas*, originally referred to as *Pseudomonas acidophila* (G-6302) and *Pseudomona mesoacidophila* (SB-72310), respectively [69, 70]. However, these compounds and other naturally occurring monobactams did not demonstrate adequate antimicrobial activity. This situation did not improve until the fully synthetic monobactam aztreonam (originally named azthreonam and later simplified) was identified and developed at Squibb in the late 1970s. This agent exhibits activity solely against Gram-negative pathogens and retains utility against organisms expressing Ambler class B zinc-dependent metallo-β-lactamases with simultaneous low allergic cross-reactivity with penicillins and cephalosporins [71]. The “az-” prefix of aztreonam refers to the azetidinone or β-lactam ring [65], while the origins of the “-tre-” (or “thre-”) portion of the generic name can be traced to the amino acid starting material threonine, used to synthesize the key 4-methyl-substituted azetidinone intermediate used in aztreonam's preparation [72] (Table 4).

## AMINOGLYCOSIDES

Aminoglycosides are a class of antibiotics originally derived from actinomycetes, particularly species of the *Streptomyces* and *Micromonospora* genera [73]. The name reflects their chemical structure, which is portmanteau of the amino sugars (“amino-”) linked by glycosidic bonds (“-glycosides”). The first aminoglycoside, streptomycin, was isolated from *Streptomyces griseus* in 1944 (“-mycin” denotes antibiotics from *Streptomyces*, while “-micin” denotes that from *Micromonospora*), followed by other agents within this class over the following decades (Table 5). The discovery of streptomycin has remained controversial because the 1952 Nobel Prize was awarded solely to Selman Waksman, whereas Albert Schatz, the graduate student who isolated streptomycin in Waksman's laboratory, was not recognized by the Nobel Committee despite later being acknowledged as a codiscoverer through legal settlement and historical review [84].

**Table 5.** Aminoglycosides

Streptomycin	The term “strepto-” refers to the twisting or coiled nature of the bacterium *Streptomyces griseus* from which it was discovered, while “-mycin” is used for antibiotics derived from this genus microorganisms.	[74]
Neomycin	The name “neomycin” is derived from the Greek prefix “neo-,” meaning “new,” and “-mycin,” a common suffix for antibiotics, indicating its origin from a *Streptomyces* species. This reflects its discovery as a new antibiotic from *Streptomyces fradiae*, distinguishing it from other previously known agents.	[75]
Kanamycin	The prefix “kana-” originates from the organism from which it was isolated, *Streptomyces kanamyceticus*, while “-mycin” indicates its origin from *Streptomyces* species.	[76]
Paromomycin	Paromomycin is derived from the genus *Streptomyces rimosus* var *paromomycinus*, from which the antibiotic was originally isolated. The original drug was named aminosidine before later being renamed.	[77]
Gentamicin	Gentamicin was discovered in 1963, and the prefix “gent-” is derived from gentian violet due to the purple color of the producing *Micromonospora purpurea* organism. “-micin” is a common suffix for antibiotics obtained from sources other than *Streptomyces.*	[78]
Tobramycin	The prefix “tobra-” is an altered form of the species from its source: *Streptomyces tenebrarius*. It is derived from the compound nebramycin, named after the state of Nebraska. The suffix “-mycin” is commonly used for antibiotics derived from *Streptomyces* species.	[79]
Netilmicin	A semisynthetic aminoglycoside: “netil-” is from *N*-ethyl, as it is an *N*-ethyl derivative of sisomicin, with a “-micin” suffix, as sisomicin originates from *Micromonospora inyoensis* (Inyo County, California).	[80]
Amikacin	A semisynthetic derivative of kanamycin: the name is believed to be a portmanteau derived from ami(noglycoside) + ka(namy)cin, reflecting its chemical structure and parent compound.	[81]
Spectinomycin	Named after the producing strain *Streptomyces spectabilis* (from Latin “spectabilis” meaning notable or spectacular), with a “-mycin” suffix.	[82]
Plazomicin	A semisynthetic aminoglycoside derived from sisomicin: the prefix was likely selected for pronounceability similar to other recently approved antimicrobials.	[83]

## MACROLIDES

This structural class was first recognized in the 1950s with the discovery of pikromycin, isolated from *Streptomyces venezuelae*. This original name reflects the bitter taste, from the Greek *pikros* for bitter [85–87]. The name “macrolide” was officially introduced by chemist R.B. Woodward during that time to describe this unique molecular architecture [88]. The term *macrolide* is derived from the compound's structure, a large ring-shaped lactone (macrolactone) that forms the antibiotic's core, usually composed of 12 to 16 atoms. Attached to this core is 1 or more sugar molecules. The word combines the Greek “makros” (large) and “-lide” (from lactone), emphasizing its large cyclic ester nature. Today, macrolides include azithromycin (an azalide: “aza-” for the nitrogen replacement and “lide” from the lactone), clarithromycin, and erythromycin (Table 6) and play an essential role in the treatment of respiratory infections as well as *Helicobacter pylori*, mycobacterial, and other infections.

**Table 6.** Macrolides

Erythromycin	Isolated from soil bacterium *Streptomyces erythreus* (now *Saccharopolyspora erythraea*) and screened from Philippine soil. The prefer “erythro-” is derived from the Greek word “erythros,” meaning “red,” due to the color of the bacterium from where it was isolated, while “-mycin” is from products from *Streptomyces* species.	[89]
Azithromycin	Semisynthetic derivative of erythromycin A with nitrogen inserted into the lactone ring to create a 15-membered azalide. The name comes from its structural similarity to erythromycin, with the “aza-” prefix indicating the nitrogen atom added to the macrolide ring and “-thromycin” suffix its similarity to erythromycin.	[90, 91]
Clarithromycin	Semisynthetic derivative of erythromycin A, which involved selective 6-*O*-methylation to enhance acid stability and pharmacokinetics. The prefix “clar-” is derived from “clarus,” meaning “clear” in Latin, reflecting its refined formulation and clearer pharmacokinetic profile vs erythromycin. The “-ithromycin” suffix indicates its classification as a macrolide antibiotic.	[92]

## QUINOLONES

Nalidixic acid, a fully synthetic 1,8-naphthyridone, was the first clinical antibacterial quinolone and was discovered in the early 1960s by George Lesher and colleagues at Sterling Drugs as a by-product during the synthesis of chloroquine [93–95]. It should be noted that a parallel discovery effort by Norman Barton and coworkers at ICI also reported antibacterial activity for the quinolone class at this time [93]. Quinolones are characterized by a bicyclic quinolone core structure, and the initial short half-life and adverse effect profile (gastrointestinal intolerance, central nervous system effects, photosensitivity, etc) limited clinical utility [96]. Early quinolone derivatives faced similar limitations until the introduction of flumequine, the first monofluoroquinolone, with the attachment of a fluorine atom at the C6 position, markedly enhancing antimicrobial activity [95, 97]. This discovery led to the development of second-generation fluoroquinolones, such as enoxacin, norfloxacin, ciprofloxacin, and ofloxacin, which exhibited extended half-lives and a broader spectrum of activity [95, 97]. “Flouro-” refers to the fluorine substituent added to the 6 position of the quinolone backbone, while the suffix “-oxacin” is a common stem for nalidixic acid derivatives, with the “ox” component perhaps arising from the exocyclic oxygen (4-oxo or ketone moiety) common to all quinolones [98]. The addition of a C7 piperazine ring in these compounds expanded activity to include other Gram-negative organisms, including *Pseudomonas* species. Ofloxacin, a later second-generation agent, was the first quinolone to demonstrate activity against certain Gram-positive organisms, such as *Staphylococcus aureus*, as well as select atypical pathogens. Subsequent third- and fourth-generation fluoroquinolones (Table 7) incorporated additional structural modifications that improved oral bioavailability, potency, safety, and efficacy against anaerobes and enhanced their Gram-positive activity.

**Table 7.** Quinolones

Ciprofloxacin	Semisynthetic derivative of norfloxacin whereby a cyclopropyl group was added. The prefix “cipro-” refers to the cyclopropyl group, while “-floxacin” is a common stem for fluorinated nalidixic acid derivatives.	[98, 99]
Ofloxacin	A synthetic racemic mixture with “o-” likely from the oxazine ring of the drug structure, with “-floxacin” a common stem for fluorinated nalidixic acid derivatives.	[100]
Levofloxacin	The active L-isomer of ofloxacin to increase potency, reduce dose, and potentially lower toxicity vs the racemic ofloxacin. “Levo-” refers to the L-isomer form of the compound, while “-floxacin” is a common stem for fluorinated nalidixic acid derivatives.	[101]
Moxifloxacin	Semisynthetic derivate with an added 8-methoxy group for enhanced Gram-positive and anaerobic coverage: “moxi-” is derived from this group.	[102]
Delafloxacin	Semisynthetic with unique anionic characteristics for enhanced activity in acidic environments. The prefix was likely selected for pronounceability similar to other recently approved antimicrobials.	[103]

## TETRACYCLINES AND GLYCYLCYCLINES

Tetracyclines are a class of antibiotics named after their core structure, consisting of 4 (“tetra”-) rings (-“cycl”) [104]. Chlortetracycline (aureomycin), the first tetracycline, was isolated by Benjamin M. Duggar in the 1940s from *Streptomyces aureofaciens*, a soil-dwelling actinomycete. The antibiotic was marketed as aureomycin, a name derived from the golden-colored mold [105, 106]. Following Food and Drug Administration (FDA) approval in 1948, chlortetracycline was introduced to the market by the Lederle Laboratories Division of American Cyanamid. By 1950, Alexander Finlay and colleagues at Chas Pfizer & Co, Inc announced the isolation of terramycin (oxytetracycline) from *Streptomyces rimosus*, obtained from soil samples in Terre Haute, Indiana [107]. The name “terramycin” likely reflects its isolation from soil (*terra*, Latin for earth) and the city from which the sample was obtained [108]. The rapid success of these antibiotics spurred extensive research leading to the development of semisynthetic or synthetic tetracycline derivatives, including the glycylcyclines (a tetracycline with a substituted glycylamido- moiety added), exemplified by tigecycline and the more recent, closely related omadacycline and eravacycline (Table 8). Successive tetracycline generations improved pharmacokinetic properties and addressed resistance. Doxycycline and minocycline provided greater bioavailability and longer half-lives, whereas newer agents such as tigecycline, omadacycline, and eravacycline were engineered to overcome common tetracycline resistance mechanisms and broaden activity against multidrug-resistant organisms.

**Table 8.** Tetracyclines and Glycylcyclines

Tetracycline	Derived from the catalytic hydrogenation of chlortetracycline, which was subsequently named tetracycline based on its chemical structure and loss of chlorine at the C7 position.	[108, 109]
Doxycycline	Semisynthetic from oxytetracycline (C6-deoxy): the name is directly derived from its chemical name, with the prefix “doxy-” a contraction of “deoxy-,” which reflects the absence of a hydroxyl group at the C-6 position.	[108, 110]
Minocycline	The name “minocycline” refers to its additional dimethylamino group at the C7 position, which is a distinguishing characteristic from prior tetracyclines.	[108, 111]
Tigecycline	Semisynthetic derivative of minocycline (C9-glycylamido) with no definitive documentation about the prefix, although it has been suggested that “tige-” originates as a partial acronymn from (bu)**ty**(l) + **g**(lycyl) + **a**(mino).	[108, 112]
Sarecycline	Semisynthetic (C7-methoxy[methyl]amino) derivate: the prefix was likely selected for pronounceability and to reflect the brand name similar to other recently approved antimicrobials.	[113]
Omadacycline	The prefix “oma-” likely refers to the aminomethyl group at the C9 position of the tetracycline core (alteration for pronounciation and marketing), although no definitive documentation exists for naming.	[114]
Eravacycline	A synthetic halogenated compound (fluorocycline) structurally related to tigecycline: the prefix was likely selected for pronounceability and to reflect the brand name similar to other recently approved antimicrobials.	[115]

## SULFONAMIDES AND ANTIFOLATE AGENTS

Sulfonamides were discovered by Gerhard Domagk in 1932 while researching antimicrobial dyes. Domagk discovered Prontosil (sulfamidochrysodine hydrochloride), a red azo dye with antibacterial properties, which was metabolized in vivo into sulfanilamide, its active component; this proved effective against murine streptococcal infections, and sulfanilamide subsequently became the first widely used antibacterial agent [116]. Several newer sulfonamide drugs (containing the sulfonamide group “-SO_2_NH_2_”) later replaced sulfanilamide, including sulfathiazole, which was used during World War II for wound infections. These discoveries were reliant on the work of George Hitchings and investigations into the metabolic inhibition of nucleic acids using purine and pyrimidine analogues, leading to the discovery of pyrimethamine, 6-mercaptopurine, and allopurinol and a shared Nobel Prize in 1988 [117]. Trimethoprim was also discovered by Hitchings and colleagues during this time and was named “tri-” after the 3-methoxy group attached to the benzene ring: meth- for the methoxy subtitutient, O- as a linking element from oxy-, and prim- shortened from pyrimidine. This agent was found to act synergistically with sulfonamides by inhibiting sequential steps in folate biosynthesis, leading to the formulation of the combination drug trimethoprim-sulfamethoxazole in 1968 (Table 9) [118, 119].

**Table 9.** Sulfonamides and Antifolate Agents

Sulfanilamide	Active metabolite of the dye Prontosil. Named as a combination of “sulfanil(ic acid)” and “-amide”	[120–122]
Sulfadiazine	Semisynthetic derivative, with name derived from sulfanilamide and with “-diazine” referring to the pyrimidine ring to which the sulfa group is attached.	[123]
Sulfamethoxazole	Derivative of other sulfa- compounds optimized for half-life: the name is a conjugation of “sulfa” + “-methoxazole” (from methoxy-isoxazole/oxazole ring structure).	[124]
Sulfacetamide	Semisynthetic derivative of sulfanilamide: “sulf(a)” + “acetamide” (*N*-acetyl derivative of sulfanilamide).	[125]
Trimethoprim	Rationally designed compound during investigation of nucleic acid inhibition with purine and pyrimidine analogous, it received its name from “tri-” after the 3-methoxy group attached to the benzene ring, “-meth-” for the methoxy subtitutient, “-o-” as a linking element from oxy-, and “-prim” shortened from pyrimidine.	[118, 119]
Pyrimethamine	Rationally designed antifolate drug initially evaluated for malaria activity, it derives its name from its chemical structure: pyrimidine + ethyl + amine.	[126]
Dapsone	Initially synthetsized as a dye/chemical, with antimicrobial activity discovered decades later. The name is derived from diamino-phenyl-sulfone, referring to its 4,4′-diaminodiphenylsulfone structure	[127]

## GLYCOPEPTIDES AND CYCLIC LIPOPEPTIDES

In the early 1950s, penicillin-resistant *S aureus* had emerged as a significant problem in hospitalized patients. Intensified efforts for new antimicrobial agents were undertaken, primarily by screening soil samples, and in 1953, organic chemist Edmund C. Kornfeld (working at Eli Lilly and Company) isolated vancomycin from a soil sample collected from the interior jungles of Borneo (sent by a missionary) containing *Streptomyces orientalis* (later reclassified as *Amycolatopsis orientalis*). The newly described antibiotic was named vancomycin (from “vanquish”) and received fast-track FDA approval, entering clinical use in 1958. Vancomycin was introduced before modern FDA efficacy requirements, resulting in continued widespread off-label use. Teicoplanin was discovered in 1978 by researchers at the Lepetit Research Center (Italy; later part of Sanofi) from the actinomycete *Actinoplanes teichomyceticus*, isolated from an Indian soil sample. It entered clinical use in Europe in 1984 but is not FDA approved in the United States. Second-generation semisynthetic lipoglycopeptides (telavancin, dalbavancin, oritavancin) were developed between the 2000s and 2010s and were designed to improve pharmacokinetics and combat emerging resistance in Gram-positive organisms (Table 10). Resistance mechanisms affecting vancomycin may also reduce susceptibility to other glycopeptides, as these agents share a common mechanism of action.

**Table 10.** Glycopeptides and Cyclic Lipopeptides

Vancomycin	Natural product isolated from soil bacterium *Amycolatopsis orientalis* (formerly *Streptomyces orientalis*) discovered in soil samples from Borneo during screening for new antibiotics against penicillin-resistant staphylococci. Vancomycin’s name was derived from the term “vanquish” and “-mycin” for antimicrobials obtained from *Streptomyces* species.	[128, 129]
Teicoplanin	Natural fermentation product from *Actinoplanes teichomyceticus* (soil actinomycete) during screening for glycopeptide analogues where it received its prefix: from “teicho” (Greek for wall, referring to cell wall target) + “planin” (coined suffix for this complex); reflects its teichoic acid-like binding.	[130]
Telavancin	A semisynthetic derivative of vancomycin with lipophilic and hydrophilic moieties for a dual mechanism, the antibiotic was named after the developing company (Theravance) and “-vancin” as a derivative of vancomycin.	[131–133]
Oritavancin	Oritavancin is a semisynthetic, long-acting lipoglycopeptide antibiotic derived from fermentation products of the bacterium *Kibdelosporangium aridum*. The etymology of the prefix is not definitively known and was likely chosen for pronounceability similar to other recently approved antimicrobials.	[134]
Dalbavancin	A precursor of dalbavancin is produced by the actinomycete *Nonomuraea gerenzanensis*. The etymology of the prefix is not definitively known and was likely chosen for pronounceability similar to other recently approved antimicrobials.	[135]
Daptomycin	Examination of soil samples from Mount Ararat in Turkey isolated the organisms producing daptomycin—the soil actinomycete *Streptomyces roseosporus*. The prefix “dap-” likely stems from decanoyl (the fatty acid tail) and amino-peptide for short, with “-mycin” a reminder of its discovery from *Streptomyces* species, although there are no verified sources for this.	[136, 137]

Daptomycin is an antibiotic based on a cyclic lipopeptide (ring-shaped peptide plus a fatty acid) that is used to treat serious Gram-positive bacterial infections, including those caused by methicillin-resistant *S aureus* and vancomycin-resistant enterococci. It was discovered in the 1980s by researchers at Eli Lilly and Company [136] but ultimately developed and commercialized by Cubist Pharmaceuticals in 1997 [138]. Daptomycin has a mechanism of action distinct from that of the glycopeptides: bacterial cell membrane depolarization rather than inhibition of cell wall synthesis. Examination of soil samples from Mount Ararat in Turkey isolated the organisms producing daptomycin—the soil actinomycete *Streptomyces roseosporus*. The origin of its name has not been published, although some have hypothesized that the prefix “dap-” refers to decanoyl (the fatty acid tail) and amino peptide for short, with -mycin a reminder of its discovery from *Streptomyces* species.

## OXAZOLIDINONES

These synthetic antibiotics were discovered in the 1970s at DuPont, but toxicity concerns (dose-limiting myelosuppression and bone marrow toxicity) stymied further development and work in this area was discontinued by DuPont in the late 1980s. The class name refers to the chemical structure of the core ring system common to the agents: 1,3-oxazolidin-2-one [139]. During subsequent exploratory investigations into the potential of oxazolidinones in the laboratory of Steven Brickner at The Upjohn Company in the late 1980s, it was discovered that structure-toxicity relationships were operative in this class [72]. This led to an expansion of the medicinal chemistry effort and ultimately analogues with an improved safety profile, good water solubility, and excellent efficacy in predictive animal efficacy models after oral or intravenous administration. Linezolid, first synthesized in 1993, emerged as the superior candidate over other agents in this class based primarily on superior pharmacokinetics [140]. Linezolid entered phase 1 clinical trials in 1995, was granted fast-track status, and was finally approved by the FDA in 2000. Linezolid's name is derived from the drug's structure, with “line-” apparently taken from the morpholine appendage and “-zolid” from the oxazolidinone ring, a consistent structural feature of this class (Table 11) [141]. The second-generation oxazolidinone tedizolid phosphate originated at Dong-A Pharmaceuticals; it was licensed by Trius Therapeutics, developed by Cubist Pharmaceuticals, and ultimately approved by the FDA in 2014 [141, 142]. Tedizolid offers incremental improvements over its progenitor linezolid in a number of areas, including once-daily dosing, a shorter treatment course, reduced hematologic toxicity, and lower monoamine oxidase inhibition, which may decrease the risk of clinically significant drug interactions. It is interesting to note the nomenclature progression leading to the final USAN-approved generic name tedizolid. TR-700 was initially assigned the name tetrezolid, reflecting the presence of the compound's tetrazole ring. When this tentative name was rejected by USAN, the name torezolid was suggested. Eventually this name was also refused, and after further discussion, the name tedizolid was finalized.

**Table 11.** Oxazolidinones

Linezolid	Fully synthetic compound, with the name derived from the drug structure, with “line-” taken from the morpholine structural component and “-zolid” from the oxazolidinone class.	[139, 141]
Tedizolid	Fully synthetic compound and similar to other “new” medications: the prefix “tedi-” was based on pronunciation preferences.	[141]

## LINCOSAMIDES

Lincosamides are named after the first antibiotic of its class, lincomycin, after Lincoln, Nebraska, where *Streptomyces lincolnensis* was first isolated in the 1960s [143]. The suffix “-amide” refers to the attached substituted prolinamide functional group, which is a key structural feature of these compounds. Discovered and developed by The Upjohn Company, clindamycin is a chlorinated semisynthetic derivative of lincomycin (“cl-” for chlorinated) designed for improved oral absorption and higher anaerobic activity [144] (Table 12).

**Table 12.** Lincosamides

Lincomycin	A natural fermentation product named after the bacteria *Streptomyces lincolnensis*, which was discovered from a soil sample in Lincoln, Nebraska, during screening for new antibiotics.	[143]
Clindamycin	Clindamycin is a chlorinated semisynthetic derivative of lincomycin (“cl-” for chlorinated) designed for improved oral absorption and higher anaerobic activity.	[144]

## RIFAMYCINS

In 1957, the department of antibiotics and natural products at Lepetiti laboratories in Milan, Italy, led by Peiro Sensi, found that an actinomycete strain, *Streptomyces mediterranei* (later reclassified as *Amycolatopsis mediterranei*), produced an antibiotic via fermentation with activity against Gram-positive bacteria [145]. Through paper chromatography, it was discovered that the antibiotic was made of 5 substances, which were named rifamycin A, B, C, D, and E, while only rifamycin B was stable enough to isolate in pure form. Sensi and colleagues at the Lepetit Research Laboratories in Milan had the habit of giving new compounds nicknames and later renaming them with names acceptable for publication. Rifamycin (initially spelled rifomycin) is named after the title of the 1955 French crime film *Rififi* [146–148], this itself from a slang term for fight/scuffle/trouble [149]. Semisynthetic derivatives of rifamycin B led to rifampin, rifabutin, rifapentine, and rifaximin (Table 13).

**Table 13.** Rifamycins

Rifampin/rifampicin	A product of *Streptomyces mediterranei* (later reclassified as *Amycolatopsis rifamycinica*): the prefix “rif-” was adopted from the title of a French film (*Rififi*). Rifampin is the US adopted name of rifampicin, named after the *N*-amino-*N*′-methylpiperazine derivative, which results in rif-amp-icin.	[146, 147, 150, 151]
Rifabutin	A derivative of rifamycin S: “-butin” is adapted from “-butyl” referring to the isobutyl side chain.	[152]
Rifapentine	Semisynthetic derivative from rifamycin SV to find a compound with a prolonged half-life. The “-pentine” suffix is derived from the cyclopentyl group added to increase lipophilicity and tissue penetration in this rifamycin derivative.	[153]
Rifaximin	A semisynthetic derivative of rifamycin SV: the “-imin” suffix is tied to the imino group that prevents significant absorption.	[154]

## POLYMYXINS

Polymyxins, including polymyxin B, were discovered in 1947 [155] and were isolated naturally from the soil bacterium originally referred to as *Bacillus polymyxa* (now *Paenibacillus polymyxa*) [156, 157]. Colistin/polymyxin E was discovered in 1949 after isolation from a similar species called *Bacillus colistinus*. This species was also referred to as *Bacillus polymyxa* var *colistinus* by Koyama et al in Japan [158] before being reclassified under the *Paenibacillus* genus. Multiple polymyxin compounds have been identified and labeled alphabetically (Table 14) [159]; however, colistin and polymyxin B were selected for clinical use due to the lowest relative risk of nephrotoxicity among the polymyxin group. Structurally, polymyxin B and colistin are cationic polypeptides with D-stereoisomers of phenylalanine and leucine, respectively [160]. The naming of polymyxins is presumably derived from their structure and the species from which they were isolated. The term “poly” refers to its polypeptide structure. The root “myx” refers to viscous or slimy exudates, associated with exopolysaccharide production by the original *B polymyxa* isolate [161].

**Table 14.** Polymyxins

Polymyxin B	The polymyxins were closely related compounds and arbitrarily labeled A-D to differentiate them, and polymyxin B was isolated from the soil bacterium originally referred to as *Bacillus polymyxa* (now *Paenibacillus polymyxa*). The prefix “poly-” refers to its polypeptide structure.	[156, 157]
Colistin (polymyxin E)	Isolated from *Bacillus colistinus*, colistin was similarly labeled polymyxin E because it was discovered 2 years after the initial polymyxins (A-D). The prefix “poly-” refers to its polypeptide structure.	[158]

## PLEUROMUTILINS

In 1951 the New York Botanical Garden was used as a garden and research facility housing numerous specimens under active investigation. Researchers at Columbia University were screening numerous fungal organisms for substances of medical interest and found that culture filtrates of 2 mushrooms, *Pleurotus mutilus* (where the class received its name) and *Pleurotus passeckerianus*, inhibited the growth of *S aureus* [162, 163]. Although only moderately active, additional work in the 1960s led to synthetic changes to the C-14 side chain to enhance potency. The first pleuromutilin, tiamulin (initially called thiamutilin for the sulfa moiety) [164], was approved for veterinary use in 1979 [164]; however, retapamulin was approved for topical use in 2007 and lefamulin in 2019 for community-acquired pneumonia. These later 2 agents maintain the “-mutilin” suffix of the class, although their prefixes were added to provide distinctive and easy-to-pronounce names, as is often done with the naming of modern compounds.

## MISCELLANEOUS ANTIBIOTICS

### Nitrofurantoin

The class of nitrofurans was developed and investigated during the 1940s, driven in part by the need for novel antibacterial agents during and after World War II. Nitrofurantoin is a synthetic compound and was discovered and developed in the late 1940s by researchers at Eaton Laboratories (a division of Norwich Pharmaceutical Company) [165]. The compound is a synthetic derivative featuring a nitrofuran ring combined with a hydantoin side chain. It was introduced into clinical use and approved by the FDA in 1953 [166].

### Fosfomycin

Discovered in 1969 through a collaborative research effort between Merck and Spain's Compañía Española de Penicilina y Antibióticos, fosfomycin was originally named phosphonomycin due to its unique epoxide-containing phosphonic acid structure but renamed due to international nonproprietary nomenclature conventions [167]. A soil sample containing *Streptomyces fradiae* was isolated from Mount Montgó near Jávea (Alicante, Spain). This isolate and others (*S viridochromogenes*, *S wedmorensis*) were screened for antimicrobial activity, and this novel phosphonic acid antibiotic was identified [168]. Fosfomycin entered clinical use in Europe in the 1970s, and an oral formulation entered use following FDA approval in 1996 in the United States.

### Metronidazole

A synthetic nitroimidazole antimicrobial agent, metronidazole was initially developed during the search for compounds with activity against *Trichomonas vaginalis*—a difficult-to-treat sexually transmitted infection at the time. In the mid-1950s, researchers at Rhône-Poulenc screened extracts from *Streptomyces* species for activity against the protozoan parasite *Trichomonas vaginalis* and identified azomycin, a naturally occurring nitroimidazole with antiparasitic activity [169]. A number of derivatives were synthesized with metronidazole, 1-(2-hydroxyethyl)-2-methyl-5-nitroimidazole, emerging as the lead compound due to improved activity and bioavailability.

The antibacterial activity against anaerobes was discovered serendipitously in 1962 [170]. A patient treated for vaginal trichomoniasis experienced concurrent resolution of necrotizing ulcerative gingivitis prompting further studies to explore its antianaerobic activity [171, 172]. Metronidazole is thus a rare drug developed primarily as an antiparasitic that later became a cornerstone for anaerobic antibacterial therapy.

### Fidaxomicin

Fidaxomicin was discovered in the mid-1970s by researchers at Abbott Laboratories while investigating the actinomycete bacteria *Dactylosporangium aurantiacum* subspecies *hamdenensis* from a soil sample collected in Hamden, Connecticut. The bacteria produced a number of compounds with activity, which were labeled alphabetically, with the most promising being tiacumicin B (“tiacum-” from the species producing the compound), an 18-membered macrocyclic lactone [173]. Initial efforts focused on the potential for staphylococcal treatment; however, oral therapy did not produce blood or tissue levels enabling further therapeutic development, and it was abandoned. It was “rediscovered” in the early 2000s with increases in *Clostridium difficile* infections and the lack of absorption leveraged for treatment [174]. Fidaxomicin maintained the suffix of the class denoting agents originating from actinomycetes (“-micin”) while the prefix follows more modern naming conventions favoring unique names and pronounceability.

### Mupirocin

Decades before Fleming's discovery of penicillin, E. de Freudenreich noticed that *Pseudomonas fluorescens* possessed the ability to prevent the growth of neighboring bacteria on culture media [175, 176]. He documented this as bacterial antagonism and it was largely forgotten until the 1960s. At that time, Beecham Pharmaceuticals (now Glaxo Smith Klein) was investigating potential antimicrobials produced by *Pseudomonas* species and found several acids when cultures were grown in liquid media (termed pseudomonic acids) [177, 178]. Pseudomonic acid A was the most abundant and most active component; however, it was metabolized quickly when attempts at development for systemic use were attempted. Yet, topical use remained a viable pathway forward. Pseudomonic acid suggested that activity was solely against *Pseudomonas* species, and the drug was renamed mupirocin with the “m-” referring to monic acid, “-piro-” an abbreviated nod to the original *Pseudomonas* isolate, and “-cin” from agents discovered from microbial sources [179].

### Chloramphenicol

The discovery of chloramphenicol was the result of a massive, global soil-screening program. In 1947, Paul R. Burkholder, a botanist at Yale University, isolated a strain of actinomycete bacteria from a soil sample collected in a mulched field near Caracas, Venezuela. *S venezuelae* was isolated and soon thereafter found to produce a compound that killed a range of pathogens, including those causing typhoid fever and Rocky Mountain spotted fever [180]. Mildred Rebstock, a research chemist at Parke-Davis, led the team that successfully synthesized chloramphenicol in the laboratory—marking the first time that a naturally occurring antibiotic was chemically generated for large-scale production [181, 182]. This significantly reduced costs allowing for mass production. Chloramphenicol received its name from the chemical structure: “chlor-” from the 2 chlorines, “-am-” for the amide group, “-phen-” for the phenyl ring, and “-icol” from the glycol side chain.

### Antifungal Agents

The development of antifungal chemotherapy agents began with the discovery of griseofulvin, a metabolic product of *Penicillium griseofulvum* in 1939 that initiated the development of modern antifungals [183] (Table 15).

**Table 15.** Antifungal Agents

Polyenes		
Nystatin	Discovered from *Streptomyces noursei*, nystatin was originally named “fungicidin” referring to its broad antifungal spectrum. It was later renamed nystatin after New York State by employees of the New York State Department of Health who discovered this agent: “ny-” (New York) + “-sta-” (state) + “-tin” (from *Streptomyces* spp).	[184, 185]
Amphotericin B	*Streptomyces nodosus* (originally M-4575) was isolated from a soil sample from the Orinoco River region of Venezuela resulting in the isolation of amphotericin A and B. The naming refers to the amphoteric chemical nature. The lipid formulations of amphotericin B retain the same compound and were developed to reduce nephrotoxicity.	[186, 187]
Natamycin	Natural fermentation product from *Streptomyces natalensis*, isolated from soil in Natal, South Africa, with the suffix “-mycin” for compounds isolated from *Streptomyces* species.	[85, 188]
**Azoles**		
Clotrimazole	A synthetic antifungal: the prefix “clo-” likely refers to the chlorophenyl functional group and “-im-” to the imidazole moiety shared with the first-generation azoles.	[189, 190]
Miconazole	A synthetic antifungal: the prefix “mico-” likely refers to mycology (from Greek *mykes* [fungus]) with “-azole” from the imidazole class.	[191, 192]
Ketoconazole	A synthetic antifungal: the prefix “keto-” refers to the ketone structure, with “-con-” Latin for with or together and “-azole” denoting the azole antifungal class.	[193]
Fluconazole	A synthetic antifungal: the prefix “flu-” refers to the fluorinated structure with “-con-” Latin for with or together and “-azole” denoting the azole antifungal class.	[194]
Itraconazole	A synthetic antifungal: there is no definitive source for the prefix, although it has been speculated that “itra-” is a variation of “tri-” for this triazole.	[195]
Voriconazole	A synthetic antifungal: it has been suggested that this trifluorinated azole received the prefix for its unique fluorination, with “vori-” an adaptation of “fluori-.”	[196]^a^
Posaconazole	A synthetic antifungal, unknown origin of the prefix “posa-.” It was likely named for pronounceability and to distinguish this agent from others in the same class.	[197]
Isavuconazole	A synthetic antifungal: newer triazole antifungals typically have a 2,4-difluorophenyl group, but isavuconazole has a 2,5-difluorophenyl group and is thus an iso(a)mer version. The prefix “isa-” was derived as shorthand for the isomer.	[198]^b^
Oteseconazole	A synthetic antifungal: unknown origin for the prefix “otese-” with the suffix a modification from other azole antifungals given the tetrazole structure. It was named for pronounceability and to distinguish this agent from others in the same class.	[199]^c^
**Echinocandins and glucan synthase inhibitors**		
Caspofungin	A semisynthetic echinocandin synthesized from pneumocandin B0: the prefix “cas-” is thought to have been derived referring to its activity against *Candida* and *Aspergillus* spp, although there is no definitive documentation for naming of this compound.	[200]
Micafungin	Developed from precursors of *Coleophoma emptetri* in 1990: “mica-“ refers to the similar prefix “myco,” meaning fungus or mushroom.	[201]
Anidulafungin	Initially identified in 1974 from echinocandin B and produced by fermentation of *Aspergillus nidulans,* where it derived its name.	[201]
Rezafungin	Structural analogue of anidulafungin in which the C5 ornithine hemiaminal is replaced with a choline amine ether. Similar to other more recently approved antimicrobials, it was likely named for pronounceability and to distinguish this agent from others in the same class.	[202]^d^
Ibrexafungerp	“Fung-” refers to the similar spectrum as echinocandins (eg, caspofungin), while the “-erp” suffix is unique to the triterpinoid class. The prefix does not have any clear origin and reflects more recent naming conventions where the drug name reflects the brand name.	[203]

^a^Pfizer, personal correspondence.

^b^Astellas, personal correspondence.

^c^Mycovia, personal correspondence.

^d^Cidara, personal correspondance.

### Polyenes

Structurally, polyenes are macrolides characterized by their macrocyclic lactone rings with multiple (“poly-”) conjugated double bonds (“-ene”) [85]. The first polyene discovered was nystatin, which was isolated from *Streptomyces noursei* in 1949 by Elizabeth Hazen and Rachel Brown [184]. Nystatin was originally named “fungicidin” [185], which presumably refers to its broad antifungal spectrum and demonstrated in vitro fungicidal activity [204]. Hazen and Brown later renamed the compound nystatin (used only topically today) after New York State, as employees of the New York State Department of Health. Unfortunately, nystatin demonstrated inadequate oral absorption and was predicted to be inefficacious for systemic invasive fungal infections. Attempts at formulating an oral suspension and intravenous formulation were unsuccessful due to concerns of vein sclerosing and infusion-related reactions with significant toxicity.

In January 1953, amphotericin compounds were discovered [205]. In exploration for antifungal agents that retained broad-spectrum activity and achieved systemic concentrations, *Streptomycete* cultures were screened for antifungal compounds. *Streptomyces nodosus* (originally M-4575) was isolated from a soil sample from the Orinoco River region of Venezuela [186], resulting in the isolation of amphotericin A and B [206]. The name amphotericin refers to the compound's amphoteric structure, which contains a carboxyl group and primary amino group on the mycosamine ring. Due to its tetraene chromophore, amphotericin B demonstrated greater antifungal activity relative to amphotericin A and became the focus for clinical use. However, amphotericin B was plagued poor oral absorption, requiring an intravenous solution with sodium deoxycholate. Significant concerns regarding the risk of nephrotoxicity and infusion-related reactions led to the development of alternative formulations. Decades later, amphotericin B lipid complex and liposomal amphotericin B were FDA approved in November 1995 and August 1997, respectively, to reduce the risk of adverse drug reactions—primarily those related to nephrotoxicity.

### Azoles

The azoles were discovered after the polyenes based on the need for therapeutic options for mycotic infections with reduced severity of infusion reactions and the dose-related nephrotoxicity associated with amphotericin B [207, 208]. The development of the azole antifungals was pioneered by the discovery of the antifungal properties of the benzimidazole moiety. The first 3 azoles marketed for clinical use were clotrimazole and miconazole in 1969 and econazole in 1974 as topical products. Later triazoles (voriconazole, posaconazole, isavuconazole) were developed specifically to enhance activity to include molds. The class suffix “azole” refers to the imidazole or triazole (3 nitrogens [“tri-”] within the azole ring) moiety that forms the structural backbone. The tetrazoles have also been developed to decrease associated toxicity and contain a 5-membered aromatic ring with 4 nitrogen atoms (“tetra-”) and 1 carbon atom. The suffix “con-azole” originates from the naming convention of miconazole and is perpetuated in the naming of other members of this class.

### Glucan Synthase Inhibitors

The echinocandins were first discovered during an assessment of fungal fermentation by-products in the 1970s. Echinocandin B was isolated from strains of *Aspergillus nidulans* var *echinulatus*, marking the first compound in this class [209]. Echinulatus is derived from “echin-,” meaning spiny or prickly and referring to the echinate structures on some *Aspergillus* species [210]. Early echinocandin compounds suffered from high toxicity, including hemolysis. Semisynthetic derivatives were developed with cilofungin (a derivative of echinocandin B) the first to enter clinical trials, and the discovery of the pneumocandins (from *Zalerion arboricola*) later developed [211, 212]. In the 1990s additional chemical modifications improved stability, solubility, and pharmacokinetics and laid the groundwork for micafungin (with key precursors from *Coleophoma empteria*) and other agents in this class, with excellent activity against most clinically relevant *Candida* species.

Ibrexafungerp is the first-in-class agent of the triterpinoid antifungals. Enfumafungin is a naturally occurring triterpene isolated in 2000 from the endophytic fungus *Hormonema carpetanum* via high-throughput screening to find novel inhibitors of the fungal glucan synthase enzyme. Enfumafungin is derived from “en-” from the endophytic fungus where it was discovered, “-fuma-” relating to *Aspergillus fumigatus*, and “-fungin” from the echinocandins that share a similar mechanism. Enfumafungin had poor stability in vivo, weak efficacy in murine models, and other problematic characteristics, and semisynthetic derivatives were pursued, with ibrexafungerp emerging as the optimal compound. “Fung-” refers to the similar spectrum as echinocandins (eg, caspofungin), while the “-erp” suffix is unique to the triterpinoids. The prefix does not have any clear origin and reflects more recent naming conventions where the drug name reflects the brand name as well.

### Other Antifungals

Flucytosine, a fluorinated analogue of cytosine, was synthesized in 1957 and designed as a fluorinated pyrimidine analogue during efforts to develop anticancer antimetabolites [213, 214]. The weak anticancer activity relegated this agent to alternative developmental pathways. By the 1960s flucytosine was repurposed as an antifungal (Table 16) [215].

**Table 16.** Other Antifungals

Flucytosine	Flucytosine, a fluorinated analogue of cytosine, was designed as a fluorinated pyrimidine analogue during efforts to develop anticancer antimetabolites.	[213, 214]
Griseofulvin	Griseofulvin derives its name directly from the scientific name of the fungus where it was originally isolated: *Penicillium griseofulvum.*	[183]
Naftifine	A synthetic antifungal discovered serendipitously: the prefix “nafti-” is derived from the naphthalene core, with “-fine” relating to its structure as an allylamine derivative.	[216, 217]
Terbinafine	Semisynthetic optimization of naftifine with a modified side chain (eg, tert-butylacetylene addition) from which it receives its prefix, with “-fine” relating to its structure as an allylamine derivative.	[217]
Butenafine	A synthetic compound: the “buten-” prefix refers to the butyl group and the butene (alkene) linker in its chemical chain, while the “-afine” suffix aligns it with its chemical cousins, the allylamines.	[218]
Tolnaftate	The name is a combination of its chemical constituents: “tol-” (from the toluene/methylphenyl group) and “-naftate” (from the naphthyl group).	[219]
Tavaborole	Discovered through screening of boron-containing compounds: the suffix is from the oxaborole structure of the drug, while “tava-” does not have a clear etymology and was likely named for pronounceability and to distinguish this agent from others	[220]
Ciclopirox	A synthetic antifungal: the prefix “ciclo-” is named after the cyclohexyl substituent, “-pir” for the pyridine ring system, and “-ox” for the hydroxy group.	[221]
Undecylenic acid	An organic 11-carbon unsaturated fatty acid derived from castor oil: “undec-” comes from the Latin *undecim* (eleven), and “-enic” refers to the double bond (alkene) in its structure.	[222]

Griseofulvin derives its name directly from the scientific name of the fungus where it was originally isolated: *P griseofulvum.* Griseofulvum is constructed by the Latin *griseus* for gray, and *fulvus* is Latin for tawny describing the color of the producing organism.

The allylamine class of antifungals (terbinafine, naftifine) are synthetic compounds discovered in the 1970s and 1980s [216]. Discovered by a combination of serendipity and targeted medicinal chemistry at the Sandoz Research Institute in Vienna, Austria, these agents [223]. The name derives from its standard term in organic chemistry, referring to a compound containing an allyl group (CH_2_ = CH-CH_2_-) attached to an amine (nitrogen atom) [224–226].

## CONCLUSION

The history and discovery of antimicrobial agents began with laboratory observations, and has advanced to the mass production of naturally occurring compounds from bacterial and fungal agents, sparking the “golden age” of antibiotic discovery. Subsequent efforts focused on high-throughput screening of compound libraries against problematic pathogens, and more modern efforts employ rational drug design combined with “multiomics” approaches. The search for new agents continues in the never-ending battle against resistance and the emergence of novel pathogenic organisms.
